# Impact of L-ornithine L-aspartate on non-alcoholic steatohepatitis-associated hyperammonemia and muscle alterations

**DOI:** 10.3389/fnut.2022.1051157

**Published:** 2022-11-16

**Authors:** Camille Pichon, Maxime Nachit, Justine Gillard, Greetje Vande Velde, Nicolas Lanthier, Isabelle A. Leclercq

**Affiliations:** ^1^Laboratory of Hepato-Gastroenterology (GAEN), Institut de Recherche Expérimentale et Clinique, Université catholique de Louvain, Brussels, Belgium; ^2^Department of Imaging and Pathology, Molecular Small Animal Imaging Center, Katholieke Universiteit Leuven, Leuven, Belgium; ^3^Service d’Hépato-Gastroentérologie, Cliniques universitaires Saint-Luc, Brussels, Belgium

**Keywords:** MAFLD (metabolic-associated fatty liver disease), NASH (non-alcoholic steatohepatitis), ammonia, myosteatosis, L-ornithine L-aspartate

## Abstract

**Background:**

Metabolic dysfunction-associated fatty liver disease (MAFLD) is the most common chronic liver disease in the world. Progression toward non-alcoholic steatohepatitis (NASH) is associated with alterations of skeletal muscle. One plausible mechanism for altered muscle compartment in liver disease is changes in ammonia metabolism. In the present study, we explored the hypothesis that NASH-associated hyperammonemia drives muscle changes as well as liver disease progression.

**Materials and methods:**

In *Alms1*-mutant mice (*foz/foz*) fed a 60% fat diet (HFD) for 12 weeks; we investigated hepatic and muscular ammonia detoxification efficiency. We then tested the effect of an 8 week-long supplementation with L-ornithine L-aspartate (LOLA), a known ammonia-lowering treatment, given after either 4 or 12 weeks of HFD for a preventive or a curative intervention, respectively. We monitored body composition, liver and muscle state by micro computed tomography (micro-CT) as well as muscle strength by four-limb grip test.

**Results:**

According to previous studies, 12 weeks of HFD induced NASH in all *foz/foz* mice. Increase of hepatic ammonia production and alterations of urea cycle efficiency were observed, leading to hyperammonemia. Concomitantly mice developed marked myosteatosis. First signs of myopenia occurred after 20 weeks of diet. Early LOLA treatment given during NASH development, but not its administration in a curative regimen, efficiently prevented myosteatosis and muscle quality, but barely impacted liver disease or, surprisingly, ammonia detoxification.

**Conclusion:**

Our study confirms the perturbation of hepatic ammonia detoxification pathways in NASH. Results from the interventional experiments suggest a direct beneficial impact of LOLA on skeletal muscle during NASH development, though it does not improve ammonia metabolism or liver disease.

## Introduction

Metabolic dysfunction-associated fatty liver disease (MAFLD) is one of the most common liver diseases, with an estimated prevalence of 25% in the adult population worldwide (1–4). The term MAFLD is defined based on the presence of hepatic fat accumulation co-occurring with either overweight or obesity, diabetes or metabolic dysregulation (5). It encompasses various disease stages, ranging from benign simple steatosis to non-alcoholic steatohepatitis (NASH) with inflammatory changes, leading to fibrosis and progressively to cirrhosis, and even hepatocellular carcinoma (1, 6).

In end-stage liver disease and cirrhosis of any etiology, loss of skeletal muscle mass and strength (also referred to as sarcopenia) confers a poor prognosis (7, 8). NASH is no exception with late-stage NASH being associated with sarcopenia (9–15). However, muscle changes are already seen at early disease stages. Recent work in animal model shows that muscle fat infiltration (also called myosteatosis) is the first visible change in skeletal muscle during the progression of MAFLD, before loss of muscle mass or muscle strength (16). Myosteatosis is specifically associated with NASH and not seen in animals with uncomplicated liver steatosis (17). Analyses of cohorts of patients support the association between severity of MAFLD and myosteatosis (17–22). Whether muscle change is the direct consequence of the dysmetabolic milieu, whether it is caused by hepatic dysfunction even when liver disease is mild or moderate or whether it is an independent process that participates to liver disease remains unknown.

One common explanation to justify altered muscle compartment in liver disease is changes in ammonia metabolism. Ammonia (NH3) is a central element in inter-organ nitrogen transport, produced during urea breakdown by intestinal bacteria, but also by glutamine deamidation and during amino acid catabolism in tissues (23–27). In homeostatic conditions, NH3 is efficiently detoxified through the urea cycle, an enzymatic machinery exclusively expressed by hepatocytes, and the glutamine synthetase (GS) pathway. In addition to being operational in the liver, GS also converts NH3 and glutamate into glutamine within other tissues such as the muscle, the brain and the kidneys (28, 29). In the muscle, glutamine is an amino acid used for protein synthesis but is also a major substrate for gluconeogenesis (30). As it represents up to 50% of the body weight, the skeletal muscle compartment becomes thus crucial to detoxify NH3 in case of liver failure (26, 31, 32).

High concentrations of NH3 have been clearly identified as a cause of neurotoxicity, leading to neurological disorders known as hepatic encephalopathy (33–35), though the mechanism is not completely understood. Ammonia is also deleterious for skeletal muscle. In animal models, excess of ammonia caused by liver insufficiency and portal hypertension negatively impacts muscle protein homeostasis *via* mechanisms implicating ATP depletion, nuclear factor-kappa B (NF-κB) activation and upregulation of myostatin, hence contributing to muscle degradation (36–40). Precarious muscle health in end-stage liver disease is at least in part due to high NH3 concentration as ammonia lowering strategies in pre-clinical models of cirrhosis alleviate sarcopenia (38). De Chiara et al. showed that hepatic ammonia detoxification pathways are impaired in pre-clinical models as well as in patients with MAFLD (41). Therefore, we propose that resulting hyperammonemia might affect skeletal muscle proteostasis also at early disease stages. The aim of our study is to evaluate ammonia metabolism in liver and muscle in a validated pre-clinical model of progressive NASH, and to test the proposition that hyperammonemia drives NASH-associated muscle alterations as well as liver disease progression. By delivering substrates that fuel the ureagenesis, the mix of amino acids L-ornithine L-aspartate (LOLA) has been shown to decrease blood ammonia levels in patients with cirrhosis, thus improving hepatic encephalopathy symptoms (42, 43). Here, we investigated the effect of LOLA in a mouse model of NASH; the *foz/foz* mice (FOZ) fed a fat rich diet.

## Materials and methods

### Animals and diets

*Alms1* mutant mice on a NOD.B10 background were bred and housed in a temperature- and humidity-controlled environment in a 12-h light/12-h dark cycle. Animals had free access to food and water at all times. At weaning (time 0), male *Alms1-/-* mice (known as fat Aussie or FOZ mice) were fed a high fat diet (HFD) (OpenSource Diets D12492; 60% of calories from fat and 0.03% cholesterol) for 12 or 20 weeks to induce NASH. Wild type (WT) littermates (*Alms1*+/+) fed a HFD or a standard rodent chow [normal diet (ND)] were used as controls. They exhibit simple steatosis and normal liver, respectively.

For the intervention experiment, FOZ mice fed a HFD received LOLA (Sigma–Aldrich) in their drinking water at a concentration adapted for a daily intake of 2 g/kg of body weight (38, 43, 44). Concentration of LOLA was calculated based on drink intake. In the prevention study, after 4 weeks of HFD mice were randomized on their body weight in two groups. The first group received LOLA-supplemented water, the second plain water for an additional 8 weeks (*n* = 4–6 per group, total duration 12 weeks HFD). In a separate study, FOZ were fed a HFD for 12 weeks to induce NASH, and then randomized on their body weight in two groups. They received LOLA-supplemented water or plain water for the last 8 weeks of the dietary experiment (*n* = 6–7 per group, total duration 20 weeks HFD). Body weight, glycemia and drink/food intake were recorded weekly. At 4 weeks intervals, we collected blood, measured muscle strength using the four-limb grip strength test (BIO-GS3, Panlab−Bioseb) and performed micro-computed tomography (micro-CT) (according to Nachit et al.; see below) (16) to monitor body composition, dorsal muscle area, spleen size, as well as skeletal muscle and liver fatty infiltration.

At the end of the experiment, 4-h fasted mice were anesthetized with ketamine/xylazine. Systemic blood was collected by cardiac puncture. Liver, duodenum and muscles (tibialis anterior, extensor digitorum longus, soleus, gastrocnemius, quadriceps, and erector spinae/quadratus lumborum) were dissected. Samples were snap frozen in liquid nitrogen and stored at −80°C until analyses, fixed in 4% formalin and embedded in paraffin (liver and muscles) or directly embedded in optimal cutting temperature compound (Tissue-Tek OCT, Sakura Finetek), and frozen for histological analyses.

Animal care and experiments were performed in accordance with European regulation, following the ARRIVE guidelines. The study protocol was approved by the university ethics committee for the use of experimental animals under the references 2016/UCL/MD/003 and 2020/UCL/MD/019.

### Micro-computed tomography

Scanning was performed on mice anesthetized with isoflurane with a Skyscan 1278 (Bruker micro-CT, Kontich, Belgium) at 50 μm voxel resolution. We used a source voltage of 65 kV and a current of 770 μA, with aluminum filter set on 1 mm (16). Exposure time was around 2–3 min per mouse. Raw images were reconstructed with the NRecon software to 3D cross-sectional image data sets. Analyses of reconstructed images were performed using SkyScan software (CTan), and segmentation of different tissue compartments was based on specific tissue density in Hounsfield units (HU). Measurements were performed as previously described (16).

Muscle and liver density were expressed as muscle or liver density (HU) to spleen density (HU) ratio. Muscle surface (in mm^2^) and density were measured on erector spinae/quadratus lumborum and psoas muscle, here designed as “dorsal muscle” at L4 and L5. Liver density was measured by placing a 3D cylindrical region of interest (ROI) (≈1.3 cm^3^) in the liver avoiding large vessels, with the mean density (HU) of the ROI volume being automatically computed. Whole-body fat volume and whole-body lean volume (muscles and organs) were also measured and reported in cm^3^. μCT-estimated fat mass and lean mass were computed by multiplying the volumetric density of fat free mass (1.05 g/cm^3^) and fat mass (0.95 g/cm^3^) multiplied by their measured volume (45).

### Histology, immunohistochemistry, and immunofluorescence

Formalin-fixed paraffin-embedded sections stained with hematoxylin-eosin (H&E) or Sirius red (SR) were used for histological evaluation of the liver and NAFLD activity score (NAS) (46), and for fibrosis assessment. NASH was defined according to the SAF algorithm (47): samples with NAS ≥ 3 and presenting at least 1 point in each sub-score (i.e., steatosis, inflammation, and ballooning) were considered as NASH while those with at least 1 point in steatosis were considered as MAFLD. Fibrosis area was automatically assessed using a dedicated software (QuPath, University of Edinburgh) and expressed as the percentage of area stained for collagen fibers on the total area of the liver section.

Immunofluorescence for GS and Carbamoyl Phosphate synthetase 1 (CPS1) was performed on 4 μm liver sections fixed in 10% formalin. Deparaffinized and rehydrated sections were submitted to heat-induced antigen retrieval in 10 mM sodium citrate buffer pH 6.0. Sections were then permeabilized in 0.3% Triton X-100, and blocked in BSA 10% + milk 3% in phosphate-buffered saline. Slides were then incubated overnight at 4°C with the following primary antibodies diluted in blocking solution: mouse anti-GS (BD Transduction Laboratories, 1:500 dilution) and rabbit anti-CPS1 (Abcam, 1:100 dilution). Secondary antibody staining was performed for 1 h at room temperature with Alexa Fluor 488 donkey anti-rabbit (Thermofisher, 1:1000 dilution) and Alexa Fluor 647 donkey anti-mouse (Thermofisher, 1:1000 dilution). Cell nuclei were stained with DAPI.

To assess muscle fibers size, wheat-germ agglutinin (RL-1022, Vector) was immunodetected on 4 μm thick paraffin sections of formalin-fixed quadriceps muscle. Immunofluorescent slides were scanned using Pannoramic 250 Flash III scanner (3DHISTECH), and the measurement of GS staining levels Author Version 2017.2 (Visiopharm, Denmark).

### Quantitative qPCR

Total RNA was extracted from liver tissue samples using Trizol reagent (Invitrogen) following the manufacturer’s recommendations. We then performed reverse-transcriptase PCR using high-capacity cDNA Reverse transcription Kit (Applied Biosystems). Amplification was carried out with SYBR Green PCR master mix (Applied Biosystems). All data are normalized using a house keeping gene (RLP19 in liver or GAPDH in muscle and duodenum) and expressed as fold-induction compared to controls arbitrarily set at 1. Primer sequences are listed in Supplementary Table 1.

### Metabolic parameters and biochemical analyses

Glucose and insulin levels were monthly monitored on tail blood from fasting mice using a glucometer (Accu-Chek) and a commercial ELISA test (Mercodia AB, Sweden), respectively.

To measure ammonia concentration, blood (120 μL) sampled in Na-heparin coated capillary tubes was rapidly centrifuged at 4°C and the plasma was collected to immediately measure ammonia concentration by an automatized colorimetric test (Fuji Dri-Chem Slide NH3-PII, Fujifilm) using dry chemistry analyzer FUJI DRI-CHEM NX500 (Fujifilm).

For muscle total lipids measurement, lipids were extracted from gastrocnemius using methanol and chloroform, and total lipids were quantitated using the vanillin–phosphoric acid reaction (48).

### Ornithine transcarbamylase activity assessment

To determine the ornithine transcarbamylase (OTC) activity, 10 μg of total proteins from mouse livers were adjusted to 100 μl with water. Samples were incubated in the presence of excess L-ornithine and carbamoyl phosphate under optimal enzyme conditions (triethanolamine solution) to trigger the reaction:


C⁢a⁢r⁢b⁢a⁢m⁢o⁢y⁢l⁢p⁢h⁢o⁢s⁢p⁢h⁢a⁢t⁢e+o⁢r⁢n⁢i⁢t⁢h⁢i⁢n⁢e→c⁢i⁢t⁢r⁢u⁢l⁢l⁢i⁢n⁢e+P⁢i

The enzymatic reaction was stopped by adding a phosphoric acid/sulfuric acid mixture (3:1). After incubation with 3% chromogenic reagent 2,3-butanedione monoxime, the rate of citrulline production was assessed spectrophotometrically at 490 nm.

### Statistics

All data are presented as mean ± SD. Statistical analyses were performed using a two-tailed Student’s *t*-test, a one-way analysis of variance (ANOVA), or a two-way ANOVA (mixed model) followed by Bonferroni’s *post hoc* using GraphPad Prism 9 software. Differences were considered significant at values of *p* ≤ 0.05.

## Results

### *Foz/foz* mice fed a high-fat diet for 12 weeks have non-alcoholic steatohepatitis and myosteatosis

**FIGURE 1:**
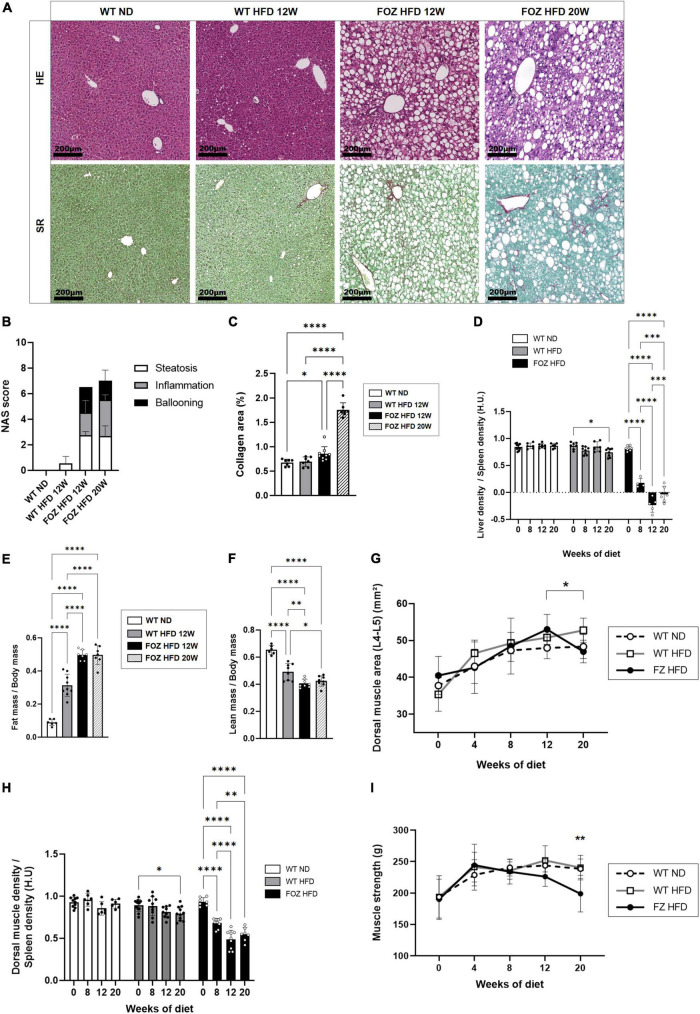
NASH is associated with skeletal muscle alterations. **(A)** Representative hematoxylin-eosin (H&E) and Sirius red (SR) staining of liver sections from wild-type normal diet (WT ND), WT high-fat fed (WT HFD), and HFD-fed fat Aussie (FOZ HFD) mice after 12 and 20 weeks of diet; **(B)** Non-alcoholic fatty liver disease activity score (NAS) (*n* = 4–9); **(C)** Collagen area assessed by digital analysis in the liver of WT ND, WT HFD and FOZ HFD after 12 and 20 weeks of HFD (*n* = 7–9); **(D)** Liver-to-spleen density measured *in vivo* by micro-CT in WT ND, WT HFD, and FOZ HFD after 0, 8, 12, and 20 weeks of diet (*n* = 4–10); **(E)** Fat mass and **(F)** fat-free mass (i.e., lean body mass including muscles and organs, bones excluded) measured *in vivo* by micro-CT (*n* = 6–9); **(G)** Dorsal muscle area (L4–L5 averaged) in WT ND, WT HFD, and FOZ HFD between 0 and 20 weeks of diet (*n* = 6–10). The * represents the significant difference between 12 W and 20 W in FOZ HFD; **(H)** Dorsal muscle-to-spleen density measured *in vivo* by micro-CT in WT ND and FOZ HFD (*n* = 6–10); **(I)** 4-limbs grip strength measured in WT ND, WT HFD, and FOZ HFD between 0 and 20 weeks of diet (*n* = 8–18). The ** represents the significant difference between FOZ HFD and the two other groups at 20 W. All data are represented as mean ± SD, *p < 0.05, **p < 0.01, ***p < 0.001, ****p < 0.0001. Statistical tests used: **(B,C,E,F)** one-way ANOVA and **(D,G,H,I)** two-way ANOVA followed by *post hoc* Bonferroni correction.

As previously reported (16, 46, 49–51), FOZ mice fed a HFD for 12 weeks are obese, and exhibit severe steatosis, inflammation and ballooning (mean NAS = 6, Figures 1A,B) with only inconspicuous fibrosis (Figures 1A,C). Inflammation and fibrosis are confirmed by gene expression analysis (S1A). At micro-CT scan examination, liver to spleen density ratio, which was close to 0.85 at the start of the feeding experiment, dropped in FOZ mice at 0.18 ± 0.09 after 8 weeks of HFD and at −0.10 ± 0.17 after 12 weeks (Figure 1D). The decrease in liver density is in agreement with the severe steatosis observed in histology. Of note, WT mice fed the HFD for the same duration have no significant change in liver phenotype through the experiment as supported by NAS < 1 (Figure 1B) and stable liver density (Figure 1D), which confirms previous results on the same model (46). Of note, spleen size was not different between mice with NASH and controls (Supplementary Figure 1B). Fat mass was increased in WT fed the HFD and much more so when FOZ were fed the HFD (Figure 1E and Supplementary Figure 1C). Concomitantly, relative lean mass was lower in WT HFD compared to ND, and even more decreased in FOZ HFD (Figure 1F). While plasma bilirubin levels were unchanged between groups (Supplementary Figure 1D), transaminases activity was elevated in FOZ HFD after 12 weeks compared to WT ND and WT HFD (Supplementary Figure 1E), supporting liver disease progression.

The size of dorsal muscle (transversally cut and examined at L4–L5) increased with time of HFD feeding in FOZ but in a similar fashion than in control WT mice fed the ND, supporting a growth effect (Figure 1G). By contrast, in accordance with previous reports (16), muscle density (as the muscle to spleen HU ratio) decreased over time in FOZ HFD mice, signing myosteatosis (Figure 1H). Muscle strength did not vary from controls up to 12 weeks of HFD feeding (Figure 1I), though it decreases at a later timepoint.

### Ammonia detoxification pathways are altered in non-alcoholic steatohepatitis livers

**FIGURE 2:**
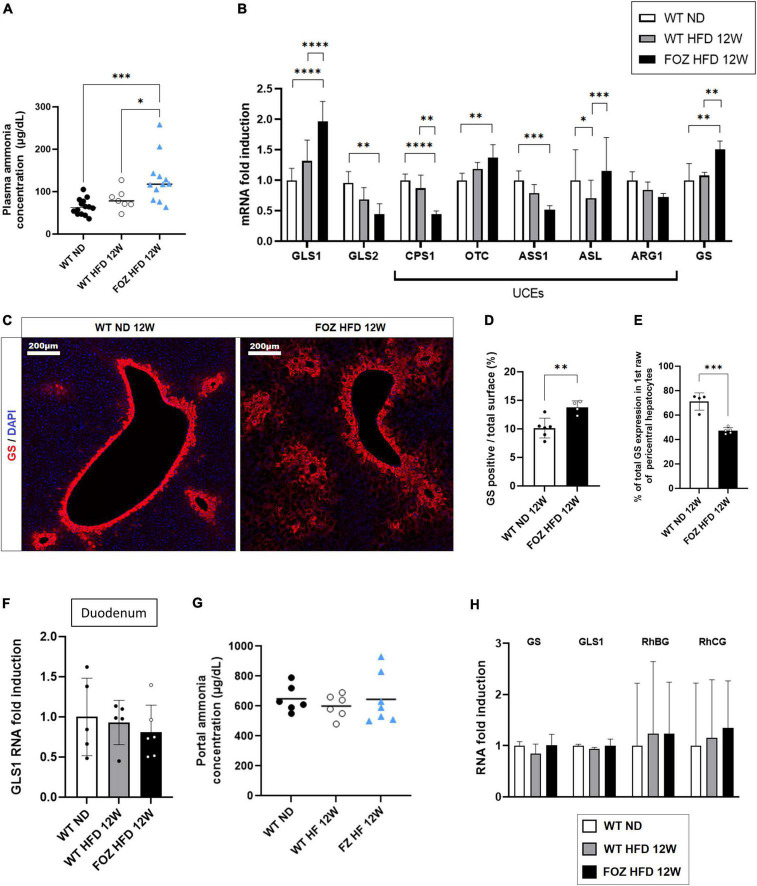
Hepatic ammonia detoxification is altered in NASH but not in simple liver steatosis. **(A)** Plasma ammonia concentrations measured in systemic blood from wild-type normal diet (WT ND), WT high-fat fed (WT HFD), and HFD-fed fat aussie (FOZ HFD) mice after 12 weeks of diet (*n* = 7–14); **(B)** mRNA levels of glutaminase (GLS) 1 and 2, of urea cycle enzymes (UCEs): carbamoyl phosphate synthetase (CPS1), ornithine transcarbamylase (OTC), Argininosuccinate synthetase (ASS1), argininosuccinate lyase (ASL), arginase (ARG1), and of glutamine synthetase (GS) in liver tissue from WT ND, WT HFD, and FOZ HFD after 12 weeks of diet (*n* = 4–9); **(C)** GS protein expression levels assessed by immunofluorescence in liver from WT ND and FOZ HFD after 12 weeks of diet; **(D)** Quantification of GS-positive tissue (in percentage) in liver sections from WT ND and FOZ HFD after 12 weeks of diet and **(E)** percentage of GS staining localized in the first raw of pericentral hepatocytes (*n* = 4–6); **(F)** mRNA levels of GLS1 in duodenum from WT ND, WT HFD, and FOZ HFD after 12 weeks of diet (*n* = 5–6); **(G)** Plasma ammonia concentrations measured in portal blood from WT ND, WT HF, and FOZ HFD after 12 weeks of diet (*n* = 6–7); **(H)** mRNA levels of GS, GLS1, and Rh Family B and C Glycoproteins (RhBG, RhCG) in muscle tissue (gastrocnemius) from WT ND, WT HFD, and FOZ HFD after 12 weeks of diet (*n* = 5–7). All data are represented as mean ± SD, *p < 0.05, **p < 0.01, ***p < 0.001, ****p < 0.0001. Statistical tests used: **(D,E)** Unpaired two-tailed *t*-test. **(A,F,G)** one-way ANOVA and **(B,H)** two-way ANOVA followed by *post hoc* Bonferroni correction.

Compared to WT ND controls, plasma ammonia levels were significantly higher in 12 weeks HFD-fed FOZ mice with NASH but not in WT fed the HFD (Figure 2A). In order to document the origin of hyperammonemia in the absence of fibrotic disease, we characterized NH3 detoxification pathways in the liver of FOZ HFD mice with NASH compared to WT HFD and WT ND controls. Hepatocytes in zone 1 and 2 (periportal) express glutaminase that generates ammonia to initiate and fuel the urea cycle. Glutaminase (GLS) 2, the liver prominent isozyme, was significantly down-regulated in NASH livers (Figure 2B). By contrast, GLS1, a highly active enzyme isoform normally poorly expressed in the liver was significantly increased by a factor 2 (Figure 2B). Among urea cycle enzymes (UCEs), the expression of carbamoyl phosphate synthetase 1 (CPS1), the rate limiting enzyme of the cycle, as well as that of argininosuccinate synthetase 1 (ASS1) were lower supporting poor urea production. Pericentral hepatocytes express GS. In NASH livers, we observed the up- regulation of GS mRNA (Figure 2B) as well as the increase of the number of GS-expressing hepatocytes (Figure 2C). Morphometrical quantification confirmed the significant increase in the relative area of GS positive cells (Figure 2D). In controls, 70% of GS expressing cells belonged to the first perivenular layer of hepatocytes while only 40% in NASH livers confirming perturbed lobular zonation (Figure 2E).

The absence of up-regulation of GLS1 in the duodenum (Figure 2F) together with similar NH3 concentration in the portal blood (Figure 2G) do not support increased NH3 production in the digestive tract. In the muscle, the expression of GS, GLS1 as well as that of ammonia transporters, Rh family B and C glycoproteins (RhBG, RhCG), was not altered (Figure 2H) supporting normal capacity for ammonia detoxification.

Altogether, higher hepatic ammonia production and lower efficiency of the urea cycle likely concur to hyperammonemia in animals with NASH.

### Preventive L-ornithine L-aspartate does not impact hepatic ammonia detoxification or liver histology but reduces myosteatosis

**FIGURE 3:**
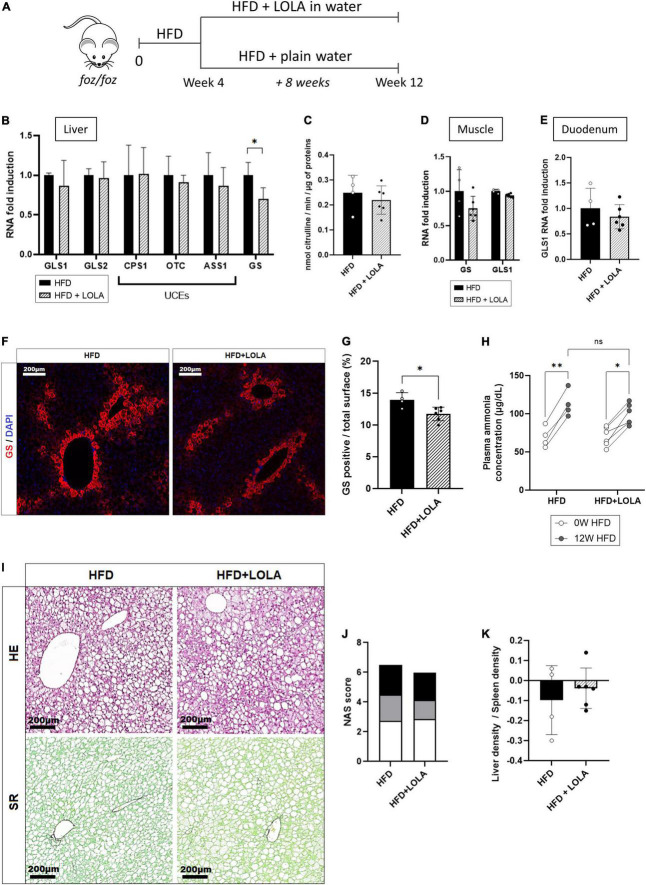
L-Ornithine L-Aspartate (LOLA) does not improve hepatic ammonia detoxification. **(A)** LOLA was administered in drinking water to HFD-fed FOZ mice (HFD + LOLA) for the last 8 weeks of the 12 weeks dietary experiment. Treated mice were compared with FOZ HFD given plain water (HFD). **(B)** mRNA levels of glutaminase (GLS) 1 and 2, of urea cycle enzymes (UCEs): carbamoyl phosphate synthetase (CPS1), ornithine transcarbamylase (OTC), Argininosuccinate synthetase (ASS1), argininosuccinate lyase (ASL), arginase (ARG1), and of glutamine synthetase (GS) in liver tissue from HFD and HFD + LOLA (*n* = 4–6); **(C)** Enzyme activity of OTC in liver tissue from HFD and HFD + LOLA (*n* = 4–6); **(D)** mRNA levels of GLS1 and GS in muscle tissue (gastrocnemius) from HFD and HFD + LOLA (*n* = 4–6); **(E)** mRNA levels of GLS1 in duodenum from HFD and HFD + LOLA (*n* = 4–6); **(F)** GS protein expression levels assessed by immunofluorescence in liver tissue from HFD and HFD + LOLA and **(G)** quantification of GS-positive tissue (in percentage) in liver sections (*n* = 4–6). **(H)** Plasma ammonia concentrations measured in systemic blood from HFD and HFD + LOLA (*n* = 4–6); **(I)** Representative hematoxylin-eosin (H&E) and Sirius red (SR) staining of liver sections from HFD and HFD + LOLA; **(J)** Non-alcoholic fatty liver disease activity score (NAS) (*n* = 4–6); **(K)** Liver-to-spleen density measured *in vivo* by micro-CT in HFD and HFD + LOLA after 12 weeks of diet (*n* = 4–6). All data are presented as mean ± SD, *p < 0.05, **p < 0.01. Statistical tests used: **(C,E,G,J,K)** Unpaired two-tailed *t*-test. **(H)** Repeated-measures two-way ANOVA and **(B,D)** two-way ANOVA followed by *post hoc* Bonferroni correction.

L-ornithine L-aspartate is a mix of stable salt of amino acids purposed to decrease ammonemia by boosting, or fueling substrates into, the urea cycle in patients with cirrhosis and hepatic encephalopathy (42). In order to test whether hyperammonemia might contribute to liver disease progression and to muscle changes, we administered LOLA in drinking water to HFD-fed FOZ mice for the last 8 weeks of the 12-weeks dietary experiment and compared them to untreated FOZ HFD (Figure 3A). Drink intake confirmed that mice received a mean LOLA daily amount of 2 g/kg of body weight with no impact on food or drink intake, on body weight gain, glycemia and insulinemia, or on liver function (Supplementary Figures 2A–G). LOLA had no effect on hepatic expression of GLS1, GLS2, or urea cycle genes (Figure 3B). In support, the activity of ornithine transcarbamylase (OTC), the second enzyme in the cycle often used to assess the efficiency of the cycle, was not changed by LOLA treatment (Figure 3C). Gene expression of GS and GLS1 in muscle (Figure 3D) and GLS1 in duodenum (Figure 3E) were not impacted either, showing that machinery for ammonia intestinal production and ammonia metabolism in muscle were not changed by LOLA supplementation. Unexpectedly, LOLA reduced hepatic GS gene and protein expression as shown by qPCR results and immunohistochemistry performed on liver tissues (Figures 3B,F,G). Altogether, LOLA barely mitigated plasma ammonia elevation in FOZ HFD (Figure 3H). The amino acid supplementation did not improve liver histology (Figure 3I) as supported by similar scores for steatosis, inflammation and ballooning (Figure 3J) and similar liver density at micro-CT scan (Figure 3K).

**FIGURE 4:**
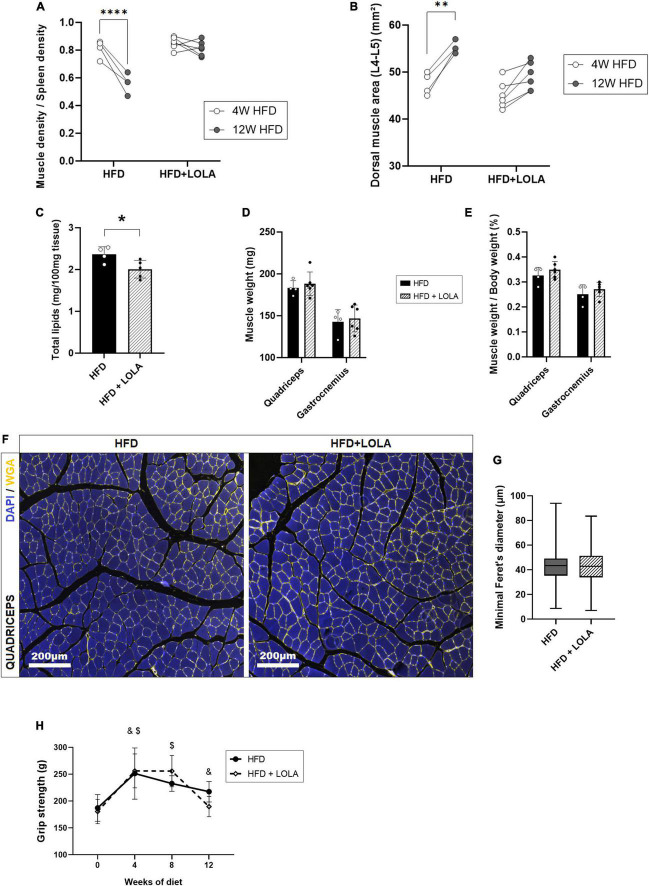
L-Ornithine L-Aspartate (LOLA) prevents myosteatosis in NASH. **(A)** Dorsal muscle-to-spleen density measured *in vivo* by micro-CT in *foz/foz* mice (FOZ) fed a high-fat diet (HFD) and given LOLA or plain water (*n* = 4–6); **(B)** Dorsal muscle area (L4–L5 averaged) measured *in vivo* by micro-CT in HFD and HFD + LOLA (*n* = 4–6); **(C)** Total lipids content measured in gastrocnemius from HFD and HFD + LOLA (*n* = 4–6); **(D)** Muscle weight and **(E)** muscle/body weight measured for quadriceps and gastrocnemius from HFD and HFD + LOLA (*n* = 4–6); **(F)** Wheat-germ agglutinin (WGA) staining of myofibers performed on paraffin-embedded quadriceps sections; **(G)** Quantification of myofibers size performed on quadriceps WGA-stained sections (*n* = 4–6); **(H)** 4-limb grip strength measured in HFD and HFD + LOLA (*n* = 4–6, repeated measures two-way ANOVA, with ^&^HFD significantly different from T0, and ^$^HFD + LOLA significantly different from T0). All data are represented as mean ± SD, *p < 0.05, **p < 0.01, ****p < 0.0001. Statistical tests used: **(C,D,E,G)** Unpaired two-tailed *t*-test. **(A,B,H)** repeated-measures two-way ANOVA followed by *post hoc* Bonferroni correction.

By contrast, LOLA prevented HFD-induced myosteatosis as it countered the drop of density measured in dorsal muscles (Figure 4A). In agreement with prevention of myosteatosis, after 12 weeks of HFD muscle area was lower in LOLA-treated animals compared to those receiving HFD only (Figure 4B). Measurement of total lipids content in the gastrocnemius confirmed lower steatosis in muscles from LOLA-supplemented mice compared to HFD (Figure 4C). The wet weight of leg muscles (quadriceps and gastrocnemius) as well as the relative muscle mass were similar between groups (Figures 4D,E). Though with a large interindividual variability, the transsectional size of muscle fibers in quadriceps was also similar between treatment groups (Figures 4F,G). The 4-legs grip strength increased during the first 4 weeks of HFD feeding, likely in relation with the growth of the animals, and then stabilized thereafter with no difference related to LOLA administration (Figure 4H).

### L-ornithine L-aspartate in a therapeutic setting did not reverse myosteatosis

As described earlier (16, 46) liver worsening toward increased fibrosis and late stages of MAFLD occurs after 12 weeks of HFD in the FOZ model. While NAS score barely increases at this stage in FOZ livers (Figures 1A,B), fibrosis area doubled between 12 and 20 weeks of HFD (Figures 1A,C), leading to a slight increase in liver density observed in micro-CT (Figure 1D). In FOZ model, this progression of fibrosis after 12 weeks of HFD occurs simultaneously with the first signs of altered muscle mass and strength. Dorsal muscle density stays unchanged suggesting no further progression of myosteatosis (Figure 1H), but muscle size decreases between 12 and 20 weeks of diet (Figure 1G). At 20 weeks of diet, muscle strength is also lower in FOZ HFD compared to WT (Figure 1I).

**FIGURE 5:**
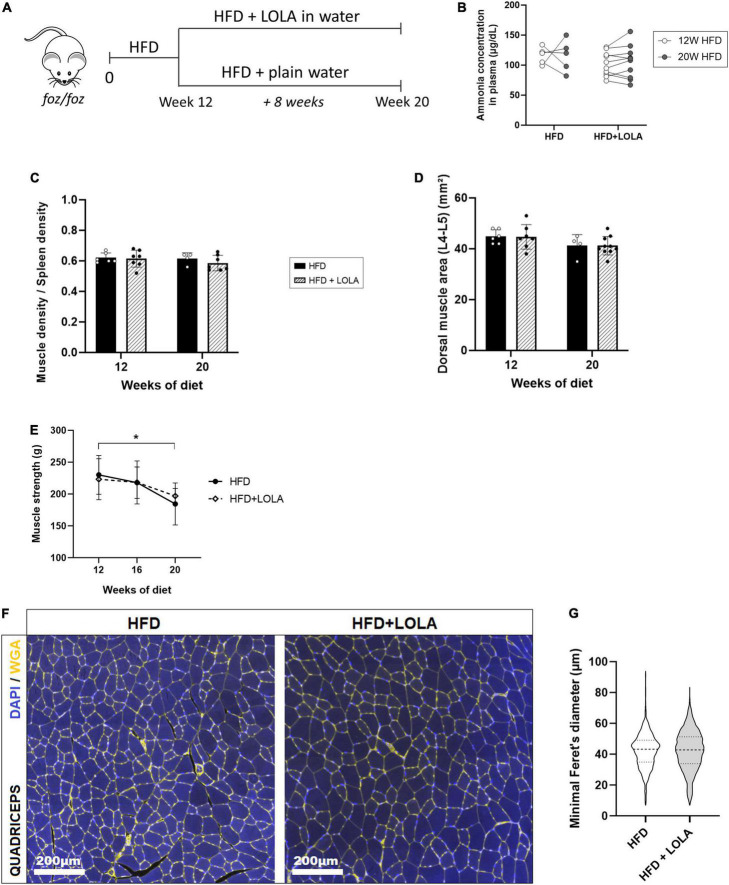
L-Ornithine L-Aspartate (LOLA) does not reverse myosteatosis in late-stage NASH. **(A)** LOLA was administered in drinking water to HFD-fed FOZ mice (HFD + LOLA) for the last 8 weeks of the 20-weeks dietary experiment. Treated mice were compared with FOZ HFD given plain water (HFD). **(B)** Plasma ammonia concentrations measured in systemic blood from HFD and HFD + LOLA after 20 weeks of diet (*n* = 5–10); **(C)** Dorsal muscle-to-spleen density measured *in vivo* by micro-CT in HFD and HFD + LOLA (*n* = 5–10); **(D)** Dorsal muscle area (L4–L5 averaged) measured *in vivo* by micro-CT in HFD and HFD + LOLA (*n* = 5–10); **(E)** 4-limb grip strength measured in HFD and HFD + LOLA (*n* = 5–10); **(F)** Wheat-germ agglutinin (WGA) staining of myofibers performed on paraffin-embedded quadriceps sections; **(G)** Quantification of myofibers size performed on quadriceps WGA-stained sections (*n* = 5–10). All data are represented as mean ± SD, *p < 0.05. Statistical tests used: **(G)** Unpaired two-tailed *t*-test. **(B–E)** Repeated-measures two-way ANOVA followed by *post hoc* Bonferroni correction.

To assess whether LOLA could prevent deterioration of muscle mass and strength during NASH progression, we administered LOLA for 8 weeks in a therapeutic setting, starting after 12 weeks of HFD feeding (Figure 5A). At this timepoint plasma ammonia was high (Figure 2A), and NASH as well as myosteatosis had developed (Figures 1B,H). LOLA had no effect on ammonemia (Figure 5B), or on liver disease. Between weeks 12 and 20, liver density slightly re-increased in FOZ HFD (Supplementary Figure 3A) likely due to progressive inflammation (Figure 1B and Supplementary Figures 3B,C) and fibrosis (Supplementary Figures 3D,E) but with no difference between animals receiving LOLA or not. Body weight gain and parameters of the metabolic syndrome (fat mass, blood glucose…) were also similar between treated and untreated groups (not shown).

Unlike what had been seen in the preventive setting, administration of LOLA in a therapeutic scheme did not reverse established myosteatosis as shown by similar muscle density between treated and untreated groups (Figure 5C). LOLA did not impact muscle size, and did not counteract loss of muscle mass and strength (Figures 5D,E and Supplementary Figures 3F,G). Fibers mean size was also unchanged by the amino acid supplementation (Figures 5F,G).

## Discussion

Muscle alterations in a context of liver disease have been described for years, and it is well known that low muscle mass and muscle fat infiltration are linked to poor prognosis in patients with cirrhosis (7, 8). Similar observations are reported in the context of NASH and NASH-related cirrhosis. Studies in patients showed muscle mass being strongly associated with NASH severity and in particular with stages of fibrosis, independently from obesity, metabolic risk factors, and insulin resistance (12, 52). The same studies also report that fibrosis and NASH prevalence is higher in patients with low muscle mass than in those without.

Intense investigation is ongoing to decipher how the interorgan communication is established in the context of MAFLD/NASH. Early phases of the liver disease in human (17, 18, 22, 53) and pre-clinical models (16) are associated with myosteatosis. In these pre-clinical models, decline in muscle strength, and eventually loss of muscle mass, are usually seen when the experiments are carried on for months and liver damages are profound (16, 54). Nachit et al. in particular conducted a longitudinal study in three different diet-induced NASH models, and showed that while myosteatosis is seen at early stages of liver disease, decrease in muscle strength and mass was only observed in a context of fibrotic NASH and not in earlier stages of the disease. Because mice with benign steatosis usually have normal muscles, we propose that the muscle changes that appear with early NASH and then intensify with fibrosis and disease progression are a consequence of the liver disease.

Hyperammonemia resulting from hepatocyte damage or metabolic dysfunction is one proposed hypothesis to explain altered muscle proteostasis in liver diseases (36, 39, 55–57). Here we describe that HFD-fed FOZ mice with hyperammonemia progressively develop fibrosing NASH, first associated with myosteatosis then with a subtle decline in muscle mass and strength. While control mice have plasma ammonia concentrations around 60–80 μg/dL, in line with values in the literature (58), we observed moderate hyperammonemia with values increasing up to 125–140 μg/dL in FOZ mice with NASH after 12 weeks of HFD feeding. Values in a similar range have previously been described in C57BL6/N mice fed Western-style diet, in C57BL6/N mice fed a high-fat choline-deficient, amino acid-defined diet and in Sprague-Dawley rats fed high-fat high-cholesterol diet (59–61).

Liver histology reveals moderate cell death, including ballooning and lipophagic granuloma, moderate inflammation, and discrete pericellular fibrosis, with no ascites or splenomegaly, i.e., a phenotype not compatible with hepatocellular failure, portal hypertension and portosystemic shunts, the usual causes of hyperammonemia. Rather, we highlight specific alterations in the nitrogen metabolism pathways in mice with NASH. The decreased expression of enzymes of the urea cycle such as the rate limiting carbamoyl-phosphate synthetase-1 and arginine-succinate synthetase 1 supports poor urea production. Urea cycle dysfunction has been repeatedly found in animal models of NASH as well as in human NASH (41, 62–64).

However, impairment of urea synthesis is not always associated with hyperammonemia whether in mice (59) or in humans (59, 64). The magnitude of the expansion of GS-positive hepatocytes and variation in net GS activity in the liver, as well as the efficiency of NH3 uptake by muscles may more or less compensate for reduced urea production. Hepatic upregulation of GLS1, a highly active enzyme that releases NH3 from glutamine, likely concurs to hyperammonemia in the FOZ model. In contrast to observations in models using western-type diet to induce NASH (59), increased intestinal glutamine catabolism and enhanced hydrolysis of urea by gut microbes do not contribute to high ammonia levels in HFD-fed FOZ mice as intestinal GLS1 transcripts and portal ammonia levels are normal.

Skeletal muscle is often considered an ammonia-scavenger organ, especially when circulating ammonia is increasing. Though GS activity is relatively low in muscle (65, 66), the relative size of the tissue compared to body mass makes it one of the main ammonia metabolizing organs (67). Net nitrogen removal by the muscle remains disputed as little is known about inter-organ flux and re-metabolization of glutamine. However, several works showed an increase in net muscle ammonia uptake and amino acid production in chronic and acute liver failure with hyperammonemia, supporting the hypothesis that skeletal muscle plays a crucial part in balancing circulating NH3 levels (68, 69). Yet, NH3 uptake by the muscle does not seem to be sufficient to maintain normal NH3 concentrations, as we observed hyperammonemia with no changes in the level of expression of muscle GS, glutaminase or NH3 transporters. As muscle morphological changes are associated with MAFLD progression, it is tempting to speculate that muscle functional disturbances limit NH3 detoxification and buffering capacity, hence contributing to hyperammonemia.

In comparison to mice with cirrhosis and myopenia which typically have plasma ammonia levels of 150–200 μg/dL or higher (70, 71), levels found in FOZ mice with NASH are more modest. It is not clear whether such moderate levels are toxic, as similar increases in ammonia concentration are observed in mice subjected to intense physical exercise (72). Nevertheless, non-cirrhotic minimal hepatic encephalopathy is a concern, even if cognitive disorders in MAFLD/NASH may be caused by factors other than systemic hyperammonemia, such as systemic inflammation, vascular dysfunction, sleep apnea, oxidative stress or gut dysbiosis, and barrier dysfunction (73–76). Behavioral and cognition tests will be of interest to evaluate cognitive performances in MAFLD/NASH models and patients as well as their evolution upon lifestyle intervention or liver-targeted therapy.

Ammonia is suggested to be involved in the pathogenesis of MAFLD. In particular, a direct profibrogenic effect of ammonia has been documented (61). Therefore, here we wanted to evaluate the effect of decreasing ammonemia on liver disease progression. LOLA is a treatment used to decrease NH3 levels in patients with hepatic encephalopathy. Surprisingly, LOLA did not restore normal ammonia levels and had no impact on liver disease progression whether in a preventive or a therapeutic scheme of administration. The mechanisms of action of LOLA are poorly understood. LOLA seems to remove ammonia *via* two distinct mechanisms: by boosting the synthesis of urea as ornithine and aspartate are substrates for argininosuccinate synthetase and ornithine transcarbamylase, and by increasing the synthesis of glutamine *via* the enzyme GS. Transcripts levels for GLS and enzymes of the urea cycle were unchanged in the liver of LOLA-treated animals. The urea cycle activity (using OCT activity as a proxy) was unchanged by treatment, and GS gene and protein expression were even decreased by LOLA. Also, muscle GS expression was not different. Though lack of LOLA efficiency has also be reported in other studies (77, 78), changes in ammonia homeostasis might be too subtle in NASH to enable visible changes due to the treatment. Indeed, while in the FOZ model ammonia concentrations are modest, in patients with cirrhosis and hepatic encephalopathy to whom LOLA is usually given as a treatment, ammonia levels are often two or even threefold higher than in healthy subjects. Wang et al. (79) recently described a positive ammonia lowering effect in C57BL/6 mice with steatohepatitis, with plasma ammonia levels as high as 340 μg/dL, which are values typically observed in cirrhosis and acute liver failure with hepatic encephalopathy (80, 81). A mouse strain effect or an issue in the assay may explain unexpectedly high ammonia values in Wang’s study as ammonemia in controls is already around a high 170 μg/dL (100 μmol/L). Conversely to our observation, LOLA almost completely cleaned steatohepatitis and fibrosis in Wang’s study. Of note, they used mice exposed to a diet rich in fat (33% of calories from fat) and containing high levels of cholesterol (2%) known to structurally and functionally damage mitochondria, alter microcirculation and promote fibrosis (82–84). Hence, there is no body weight gain in the face of energy overload, the qualification of the model as “sarcopenic obesity” being, to say the least, misleading (79). Rather than ammonia removal, hepatoprotection could also be related to an antioxidant effect due to the conversion into glutathione of LOLA-derived glutamate or to improved microcirculation due to nitric oxide formation from L-arginine. In fructose induced MAFLD, supplementation with amino such as citrulline or methionine positively modulate steatosis, fibrosis and inflammation likely by improving insulin sensitivity, lipid metabolism an oxidative stress (85, 86).

Matching the perturbed expression of genes and proteins that govern hepatic nitrogen conversion, hepatic accumulation of ammonia has been found in patients with MAFLD/NASH (64, 87). Impairment of hepatic nitrogen clearance was also measured in patients with MAFLD (63). To the best of our knowledge, there is no report on elevated blood ammonia levels in large cohorts of patients with MAFLD/NASH. Although difficult to anticipate in the light of the results of the present study, clinical trials are awaited to decide whether, if hyperammonemia is confirmed, ammonia lowering therapy may favorably influence the outcome of NASH.

*Foz/foz* HFD mice receiving preventive LOLA supplementation showed remarkable resistance to the development of myosteatosis and an overall better muscle quality, as supported by a muscle strength similar to that of untreated animals, despite smaller muscles. L-ornithine and LOLA are commonly used to increase athletic performance and have a beneficial impact on muscle protein homeostasis in patients with cirrhosis and hepatic encephalopathy (38, 42). It is thus probable that the treatment has direct beneficial effect on the muscles in the FOZ model as well. The mechanism at play is unknown but does not reflect a global reduction of ectopic fat as LOLA had no effect on body weight gain, global fat mass, liver fat nor corrected the dysmetabolic profile. LOLA treatment prevented fat accumulation in the skeletal muscle compartment, without improving plasma NH3 concentrations nor rebalance hepatic nitrogen metabolism, hence invalidating the aforementioned hypothesis that myosteatosis may impair the ability of muscles to detoxify NH3.

Though over the years, a strong rationale has built-up to incriminate altered hepatic nitrogen conversion and hyperammonemia in the pathogenesis of NASH and of muscle alterations that worsen NASH prognosis, evidence is still missing to designate amongst urea cycle, ammonia production versus scavenging and ammonia levels, which one would be an appropriate therapeutic target to treat NASH.

## Data availability statement

The original contributions presented in this study are included in the article/Supplementary material, further inquiries can be directed to the corresponding author.

## Ethics statement

This animal study was reviewed and approved by the Université catholique de Louvain Ethical Committee.

## Author contributions

CP, MN, and IL conceived and designed the study. CP and MN performed the *in vivo* experiments, prepared the material, performed biochemical, and histological and gene expression analyses. CP, MN, and GV acquired and analyzed micro-CT data. CP, MN, JG, NL, and IL critically reviewed and analyzed the data. JG and IL wrote the manuscript. All authors contributed to the article and approved the submitted version.

## Abbreviations

ARG1: arginase 1
ASL: argininosuccinate lyase
ASS1: argininosuccinate synthetase 1
CPS1: carbamoyl phosphate synthetase 1
FOZ: *foz/foz* mice
GLS: glutaminase
GS: glutamine synthetase
H&E: hematoxylin-eosin
HFD: high-fat diet
HU: Hounsfield units
LOLA: L-ornithine L-aspartate
MAFLD: metabolic dysfunction-associated fatty liver disease
NAS: non-alcoholic fatty liver disease activity score
NASH: non-alcoholic steatohepatitis
ND: normal diet
NF-κB: nuclear factor-kappa B
NH3: ammonia
OTC: ornithine transcarbamylase
RhBG: Rh family B glycoproteins
RhCG: Rh family C glycoproteins
ROI: region of interest
SAF: steatosis-activity-fibrosis
SR: Sirius red
UCE: urea cycle enzyme
WT: wild-type.
