# Supplementary material for: Impact of L-ornithine L-aspartate on non-alcoholic steatohepatitis-associated hyperammonemia and muscle alterations
Source: Front Nutr. 2022 Nov 16;9:1051157. doi: 10.3389/fnut.2022.1051157 (PMC9709200; doi:10.3389/fnut.2022.1051157)
Supplement: Supplementary file 1 [file Table_1.docx]

| **Gene** | **5' primer** | **3' primer** |
| --- | --- | --- |
| F4/80 | GATGAATTCCCGTGTTGTTGGT | ACATCAGTGTTCCAGGAGACACA |
| MCP1 | CCACTCACCTGC TGCTACTCAT | CTGCTGGT GATCCTCTTGT |
| Coll1a | GACTGGAAGAGCGGAGAGTACTG | CAGGTCTGACCTGTCTCCATGTT |
| gls1 | CAACGTCAGATGGTGTCATGCT | CAGCAACCTTCCCTCCAGAC |
| gls2 | CTCTTCCAAAAGTGTGTGAGC | CCGTGAACTCCTCAAAGTCAGG |
| cps1 | CACCAATTTCCAGGTGACCA | TACTGCTTTAGGCGGCCTTT |
| otc | AGGGTCACACTTCTGTGGTTC | CAGAGAGCCATAGCATGTACTG |
| ass1 | CATTGGAATGAAGTCCCGAG | GATTTTGCGTACTTCCCGAT |
| asl | TGATGCCCCAGAAGAAAAAC | TTTGCGGACCAGGTAGTAGG |
| arg1 | ACAAGACAGGGCTCCTTTCAG | TGAGTTCCGAAGCAAGCCAA |
| glul | GCTGCAAGACCCGTACCCT | TTCCACTCAGGTAACTCTTCCACA |
| rhbg | GCCTGCAGAGTGTGTTTCCA | GAGCTGATACACGGCCTGAGA |
| rhcg | GGATACCCCTTCTTGGACTCTTC | TGCCTTGGAACATGGGAAAT |

**Table S1:**

List of primers used for RT-qPCR
