# Supplementary figures and images for: Impact of L-ornithine L-aspartate on non-alcoholic steatohepatitis-associated hyperammonemia and muscle alterations
Source: Front Nutr. 2022 Nov 16;9:1051157. doi: 10.3389/fnut.2022.1051157 (PMC9709200; doi:10.3389/fnut.2022.1051157)

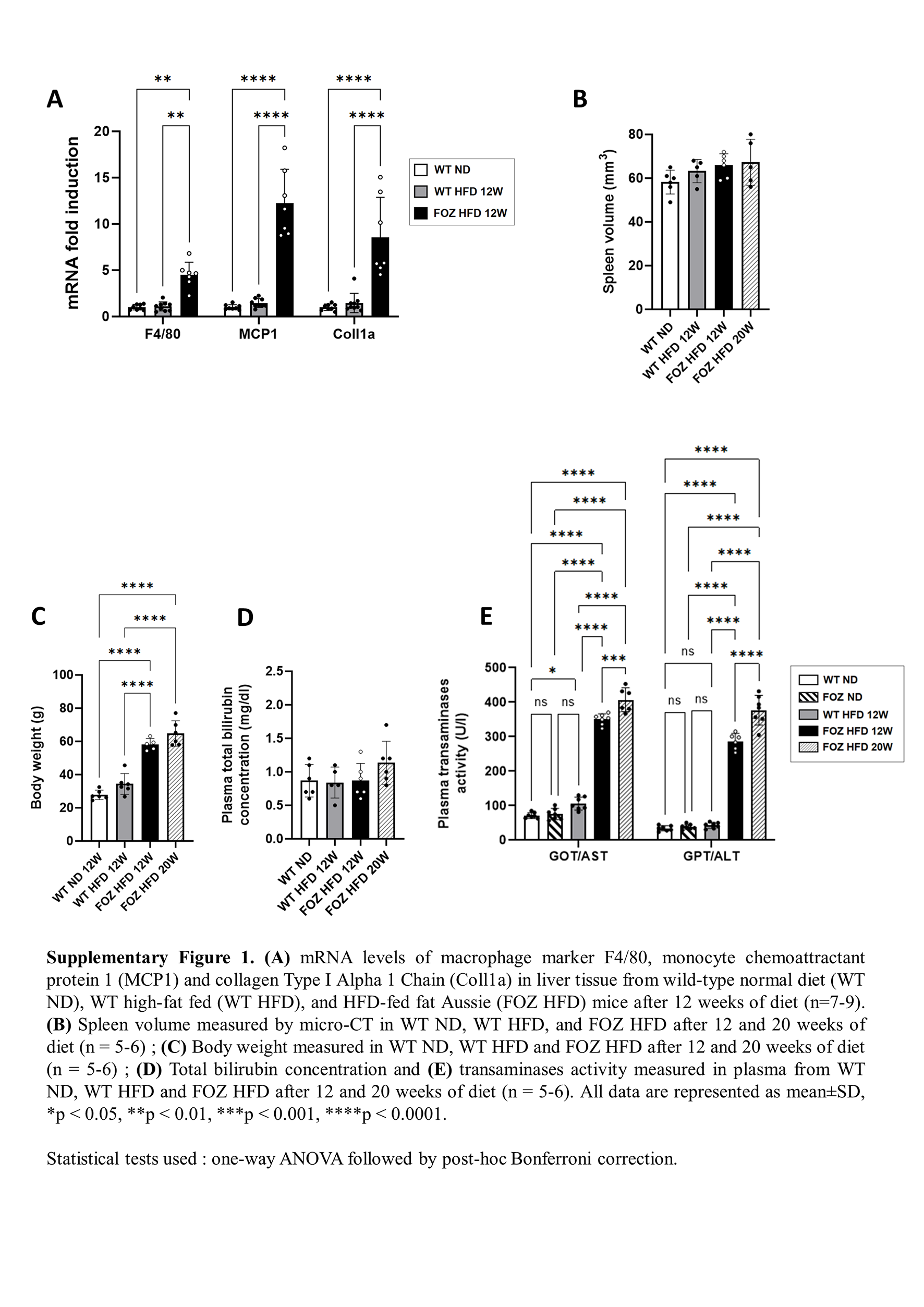

Supplement: Supplementary file 2 [file Image_1.TIF]

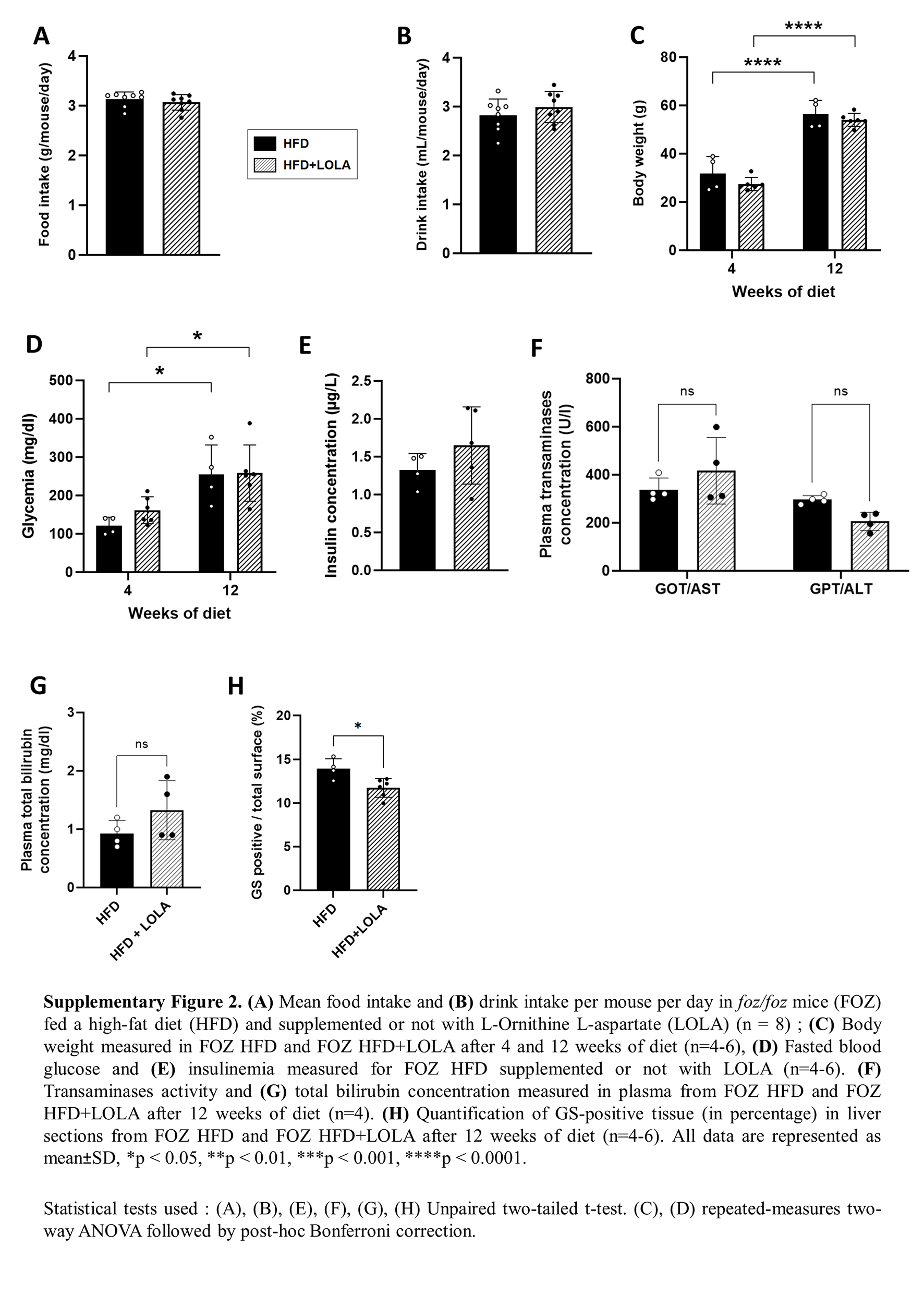

Supplement: Supplementary file 3 [file Image_2.tif]

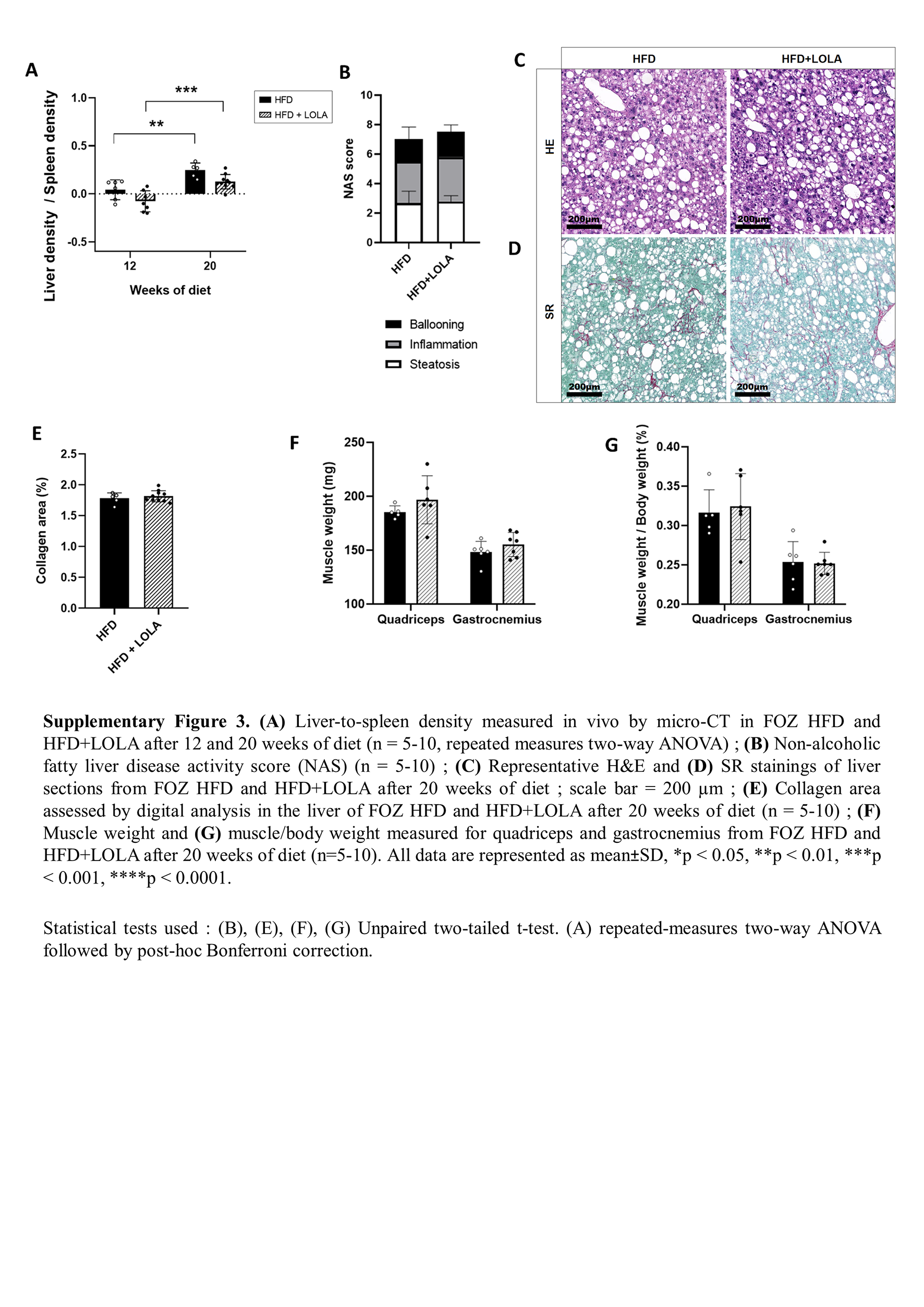

Supplement: Supplementary file 4 [file Image_3.TIF]
